# Assessment of Safety and Errors in Laparoscopic Cholecystectomy in the Treatment of Gallstone Disease in Southeastern Mexico

**DOI:** 10.3390/jcm15134869

**Published:** 2026-06-23

**Authors:** Zyanya Patricia Alvarez Tiburcio, Kevin David Gonzalez Gomez, Hector Ricardo Ordaz Alvarez, Jose Luis Vargas Basurto, Alfonso Gerardo Perez Morales, Juan Carlos Castellanos Juarez, Octavio Avila Mercado, Miguel Angel Carrasco Arroniz, Jose Luis Suarez Alvarez, Gabriela Virgen Rosario, Zaira Eunice Montes Osorio, Jorge Sempe Minvielle, Rafael Hernandez Espinoza, Ana Delfina Cano Contreras, Federico Bernhardo Roesch Dietlen

**Affiliations:** 1 Digestive Physiology and Motility Laboratory, Biological and Medical Research Institute, Universidad Veracruzana, Veracruz 91700, Mexico; zyanypaty@hotmail.com (Z.P.A.T.); david.12.kg@gmail.com (K.D.G.G.); hector.o.alvarez56@gmail.com (H.R.O.A.); jose_luis_3011@hotmail.com (J.L.V.B.); anacano_143@hotmail.com (A.D.C.C.); 2 Faculty of Medicine, Veracruz Region, Universidad Veracruzana, Veracruz 91700, Mexico; aperezm3@hotmail.com (A.G.P.M.); zmontes@uv.mx (Z.E.M.O.); 3 Surgical Department, Hospital Español, Veracruz 91700, Mexico; castellanosjuarez@yahoo.com.mx (J.C.C.J.); suarez_alvarez@hotmail.com (J.L.S.A.); rafael2@rocketmail.com (R.H.E.); 4 Surgical Department, Hospital Milenium, Boca del Río 94299, Mexico; droctavioavila@yahoo.com.mx (O.A.M.); drcarrascom@hotmail.com (M.A.C.A.); 5 Surgical Department, High Specialty Hospital of the Ministry of Health, Veracruz 91700, Mexico; gabrielavirgen271@gmail.com (G.V.R.); jorge.sempem@hotmail.com (J.S.M.)

**Keywords:** laparoscopic cholecystectomy, OCHRA, surgical errors, parkland severity, critical view of safety

## Abstract

**Background/Objectives**: The Observational Clinical Human Reliability Assessment (OCHRA) system evaluates surgical performance by identifying intraoperative errors, yet evidence on error patterns and procedural safety in laparoscopic cholecystectomy (LC) remains limited. This study aimed to assess LC safety using established parameters and to describe intraoperative errors through the OCHRA system in patients with gallstone disease in Veracruz, Mexico. **Methods**: An observational, retrospective, analytical study was conducted between January 2022 and March 2025. Surgical videos from 11 surgical teams were reviewed. Intraoperative errors were classified using the OCHRA system across the three key steps of LC, while procedural safety was assessed through achievement of the Critical View of Safety (CVS) using the Doublet Photographic Score (DPS). Comparisons were performed according to the Parkland Grading Scale. Statistical analysis was conducted using SPSS version 26. **Results**: A total of 106 patients were included (67% women; mean age 45 ± 13 years; BMI 25.1 ± 3.2 kg/m^2^). Total LC was performed in 95% of cases and subtotal LC in 5%. Parkland grade 3 was the most frequent (32.1%). Overall, 3180 operative steps were evaluated, and 705 errors (22.1%) were identified. Procedural errors predominated across all phases (97–99%), mainly due to step repetition or additional steps, whereas execution errors were uncommon (1–3%). A satisfactory CVS was achieved in 54.7% of cases. No bile duct injuries were observed. **Conclusions**: The OCHRA system enabled detailed the identification of intraoperative error patterns and their relationship with surgical difficulty. Higher anatomical severity was associated with increased procedural errors and lower rates of adequate CVS achievement. These findings support structured video-based performance assessment as a complementary tool to established safety principles, with the potential to guide targeted training and improve surgical consistency in laparoscopic cholecystectomy.

## 1. Introduction

Laparoscopic cholecystectomy (LC) is one of the most commonly performed surgical procedures worldwide and remains the treatment of choice for symptomatic gallstone disease. Despite its widespread use, LC is not free of complications, with reported rates ranging from 0.5% to 6% and an associated mortality of 0.1–1% [1,2]. The most serious complication is bile duct injury, occurring in 0.3% to 0.7% of cases [3,4,5,6], which is associated with significant morbidity, reduced quality of life, increased healthcare costs, and potential medico-legal consequences [7,8,9].

To improve surgical safety, the Society of American Gastrointestinal and Endoscopic Surgeons (SAGES) introduced the Safe Cholecystectomy Program, emphasizing standardized strategies to reduce bile duct injury. Among these, the Critical View of Safety (CVS) remains the cornerstone of safe cholecystectomy, ensuring correct identification of anatomical structures before transection. Additional approaches include intraoperative severity stratification using the Parkland Grading Scale (PGS), intraoperative imaging techniques, and the adoption of bailout procedures such as subtotal cholecystectomy or conversion to open surgery when necessary [5,10,11,12,13].

Furthermore, a growing body of contemporary research has focused on the implementation of artificial intelligence (AI) models for the intraoperative identification of key anatomical landmarks. While these approaches aim to enhance surgical safety and serve as an adjunct to established principles, current evidence suggests that the performance and reliability of such AI-driven systems remain limited in more complex operative scenarios, particularly in cases with higher Parkland grades, where inflammation and distorted anatomy pose significant challenges [14].

While these strategies primarily address anatomical identification and procedural safety, they do not directly assess the technical and cognitive performance of the surgeon during the operation. In this regard, increasing attention has been directed toward objective methods for evaluating surgical performance and human factors associated with adverse events. The Observational Clinical Human Reliability Analysis (OCHRA) system is a structured framework that enables the identification and classification of intraoperative errors, distinguishing between procedural and execution errors, and providing insight into performance variability during surgical tasks [15,16,17].

However, it is important to recognize that OCHRA is not a direct measure of surgical safety, but rather a tool for performance assessment that may complement established safety principles such as CVS. Despite its potential utility, evidence regarding its application in laparoscopic cholecystectomy, particularly in real-world clinical settings and in Latin American populations, remains limited [18,19,20].

Therefore, the aim of this study was to evaluate the safety of laparoscopic cholecystectomy using established safety parameters and to describe intraoperative error patterns through the OCHRA system in patients with gallstone disease treated in southeastern Mexico.

## 2. Materials and Methods

Study design. A retrospective, observational, analytical study was conducted in patients who underwent laparoscopic cholecystectomy (LC) between January 2022 and March 2025 in Veracruz, Mexico.

Study population and inclusion criteria. Patients aged 18 to 80 years with a confirmed diagnosis of gallstone disease were retrospectively enrolled from surgical video records obtained from 11 surgical teams, all consisting of general surgeons practicing in private hospitals. Patient selection was performed independently of whether the procedure was elective or emergency, as well as regardless of intraoperative severity according to the Parkland Grading Scale. Inclusion criteria were: patients aged 18 to 80 years, a confirmed diagnosis of gallstone disease, and completion of laparoscopic cholecystectomy during the study period. Exclusion criteria included procedures converted to open surgery and incomplete or inadequate video recordings that did not allow for proper visualization of the critical operative steps.

Video assessment. All surgical videos were reviewed and analyzed by trained expert surgeons with experience in laparoscopic cholecystectomy and familiarity with the OCHRA methodology. Evaluations of the Critical View of Safety (CVS) and intraoperative errors were performed independently. To minimize assessment bias, the surgeons who performed the procedures were excluded from the evaluation process of their own videos.

Variables analyzed. The variables collected included demographic and anthropometric characteristics, including age, sex, and body mass index (BMI), as well as associated comorbidities, type of cholecystectomy performed, total operative time, and duration of the main procedural steps, including port insertion, cystic plate dissection, clipping and division of the cystic duct and artery, and gallbladder dissection from the hepatic bed. In addition, intraoperative findings, intraoperative complications, and postoperative complications were recorded.

Surgical teams. Information regarding the participating surgeons was also collected, including age, sex, years of surgical experience, and average monthly surgical volume.

Severity assessment. Patients were classified into five groups according to the Parkland Grading Scale (PGS), which allows intraoperative assessment of disease severity, predicts surgical difficulty, and provides guidance for intraoperative decision-making aimed at preventing bile duct injury [10,13].

Critical View of Safety (CVS). The CVS was assessed according to the Strasberg criteria using the Doublet Photographic Score (DPS) described by Sanford and Strasberg [21]. This score evaluates still images of the operative field across three key domains: visualization of two structures entering the gallbladder, exposure of the cystic plate, and clearance of the hepatocystic triangle. Each domain was graded on a three-level scale, assigning 2 points when the criterion was clearly fulfilled, 1 point when the finding was visible but suboptimal or required careful interpretation, and 0 points when the criterion could not be reliably confirmed. The total DPS ranges from 0 to 6, and a score ≥5 was considered indicative of satisfactory CVS achievement.

OCHRA error analysis. Intraoperative errors were identified and classified using the Observational Clinical Human Reliability Analysis (OCHRA) system, as described by Tang et al. [16]. This system distinguishes between procedural errors and execution errors. Procedural errors were defined as deviations in the expected sequence or completion of operative steps, including omission, partial completion, repetition, addition of an unnecessary step, substitution of one step for another, or performance of a step out of sequence. Execution errors were defined as deviations in the technical performance of a step, including excessive or insufficient force, speed, depth, distance, duration, or rotation; incorrect orientation, direction, or spatial location; and performance of a maneuver on the wrong object or surgical plane.

Three key operative phases of LC were evaluated: cystic plate dissection, clipping and division of the cystic duct and artery, and gallbladder dissection from the hepatic bed. Based on this assessment, total error scores were calculated, including global errors, procedural errors, and execution errors.

Statistical analysis. Descriptive and inferential statistical analyses were performed using IBM SPSS Statistics for Windows, version 26.0. Quantitative variables were expressed as means with standard deviation or medians with interquartile ranges, as appropriate, while qualitative variables were reported as absolute frequencies and percentages. Data distribution was assessed using the Kolmogorov–Smirnov test, and homoscedasticity was evaluated using Levene’s test. For comparisons among three or more groups, one-way analysis of variance (ANOVA), with Tukey HSD or Games–Howell post hoc tests, was used for normally distributed data. When the assumption of normality was not met, the Kruskal–Wallis test with Dunn post hoc analysis was applied. Correlations were analyzed using Spearman’s rank correlation coefficient. Categorical variables were compared using the χ^2^ test or Fisher’s exact test, as appropriate. A *p* value < 0.05 was considered statistically significant.

## 3. Results

### 3.1. Case Selection

**Figure 1:**
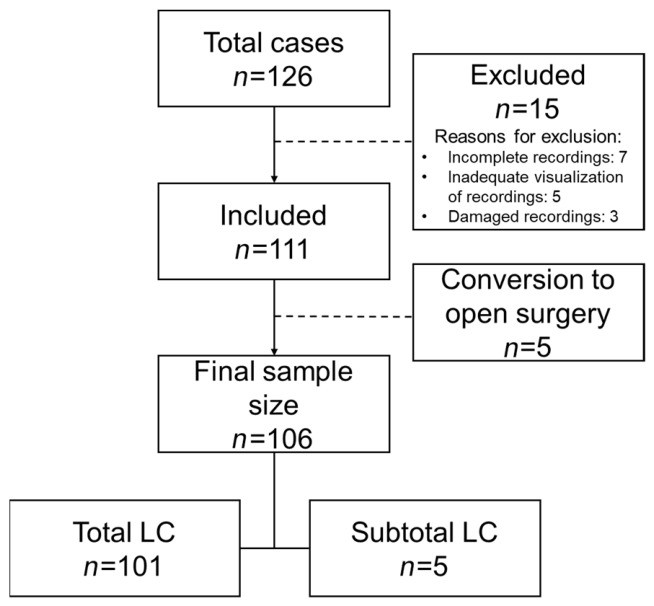
Case selection and distribution. LC: laparoscopic cholecystectomy.

A total of 126 patients were initially included; however, 15 were excluded, resulting in a final study population of 111 cases. LC was converted to open surgery in 5 cases (4.7%). Among the remaining 106 procedures completed laparoscopically, total cholecystectomy was performed in 101 cases (95.3%) and subtotal cholecystectomy in 5 cases (4.7%) (Figure 1).

### 3.2. Surgeon Profile

The 106 LC were performed by 11 different surgical teams. The surgeons had a mean age of 52 ± 10 years; 81.8% were men, and 36.3% had more than 10 years of surgical experience. Surgeons reported performing 4–9 cases per month in 45.5%, followed by ≥10 cases in 36.3% and 1–3 cases in 18.2%.

### 3.3. Patient Characteristics and Comorbidities

The mean age of the patients was 45.9 ± 13.6 years (range 19–78), with a predominance of women (67.9%) and a mean BMI of 25.1 ± 3.29 kg/m^2^. Associated comorbidities included obesity in 18 patients (16.98%), systemic arterial hypertension in 15 (14.16%), diabetes mellitus in 3 (2.83%), and dyslipidemia in 2 (1.89%). Excessive alcohol consumption was reported in 19 patients (17.9%), smoking in 11 (10.4%), and 30 patients (28.3%) had a history of previous abdominal surgery.

### 3.4. Severity Classification

Patients were stratified into five groups according to the PGS: Grade 1, 30 cases (28.3%); Grade 2, 25 cases (23.6%); Grade 3, 34 cases (32.1%); Grade 4, 14 cases (13.2%); and Grade 5, 3 cases (2.8%).

### 3.5. Operative Time

The mean total operative time was 53.69 ± 24.47 min, with a mean of 5 min for port placement, 17.62 min for cystic plate dissection, 7.68 min for clipping and ligation of the cystic artery and duct, and 11.25 min for gallbladder dissection from the hepatic bed. Cystic plate dissection and gallbladder dissection times were shorter in Grades 1 and 2 and increased substantially in Grades 3, 4, and 5 as shown in Table 1, with statistically significant differences between groups (*p* < 0.001). The time required for clipping and ligation also increased with the grading but without reaching statistical significance (*p* = 0.154).

### 3.6. Intraoperative Complications and Additional Procedures

**Figure 2:**
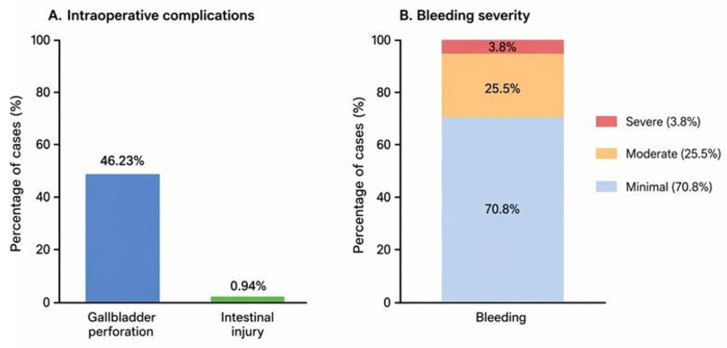
Intraoperative complications and bleeding severity during laparoscopic cholecystectomy. Panel (**A**) shows the proportion of intraoperative complications. Gallbladder perforation was the most frequent complication, while intestinal injury was rare. No bile duct injuries were recorded. Panel (**B**) illustrates the distribution of bleeding severity, categorized as minimal, moderate, and severe. Note: Percentages are calculated based on the total number of cases (*n* = 106).

The most frequent intraoperative complications were gallbladder perforation and bleeding, predominantly moderate to severe severity. Only one case of intestinal injury was recorded, and no bile duct injuries were observed. Intraoperative cholangiography was performed in four patients. An external drain was placed in 22.64% of cases, with statistically significant differences observed between Parkland Grading Scale (PGS) groups (*p* < 0.0001). The distribution of intraoperative complications and bleeding severity is shown in Figure 2.

### 3.7. Critical View of Safety of the Cystic Artery and Duct (CVS)

The DPS based on three predefined criteria showed the following results: (1) Exposure of the cystic plate was clearly visible in 53.8% of cases (*n* = 57), partially visible in 38.7% (*n* = 41), and not visible in 7.5% (*n* = 8); Exposure of the hepatocystic triangle was clearly visible in 55.7% (*n* = 59), partially obscured in 34.9% (*n* = 37), and obscured by tissue in 9.4% (*n* = 10). Visualization of two structures entering the gallbladder was clearly visible in 81.1% (*n* = 86), visible but overlapping in 11.3% (*n* = 12), and only one or nonvisible in 7.5% (*n* = 8). A satisfactory CVS, defined as a DPS ≥ 5, was achieved in 54.7% of cases (*n* = 58). The exposure of the structures and DPS according to the PGS are summarized in Table 2.

The DPS correlated negatively with the severity grade (r = −0.568, *p* = <0.001). Significant differences were observed between severity grades and DPS scores using the Kruskal–Wallis test (*p* < 0.001). Post hoc analysis revealed lower scores in higher severity grades: Grade 5 vs. Grade 1 (*p* = 0.018), Grade 4 vs. Grade 2 (*p* = 0.011) and Grade 1 (*p* < 0.001), and Grade 3 vs. Grade 2 (*p* = 0.012) and Grade 1 (*p* < 0.001). The DPS values for each severity group are detailed within the same table.

### 3.8. Identification and Categorization of Procedural and Execution Errors Using OCHRA

A total of 3180 surgical steps were evaluated, of which 705 errors (22.1%) were identified. During cystic plate identification and dissection, 231 errors (7.2%) occurred; during clipping and division of the cystic artery and duct, there were 237 errors (7.4%); and during dissection of the gallbladder from the hepatic bed, there were 237 errors (7.4%).

During cystic plate dissection, procedural errors accounted for 97.2% of errors, while execution errors represented 3.8%. For clipping and division of the cystic artery and duct, procedural errors accounted for 96.2% and execution errors for 1.9%. Finally, during gallbladder dissection from the hepatic bed, procedural errors comprised 96.2% and execution errors 5.7%. Across all operative steps, the most prevalent errors were procedural, particularly those involving the repetition of a step or the performance of an additional, unnecessary step (Table 3).

### 3.9. Correlation Between Severity and OCHRA Errors

Severity grade correlated positively with the total number of procedural errors (r = 0.439, *p* < 0.001). No statistically significant correlation was found for execution errors across the three operative steps. Post hoc analysis for the total procedural errors revealed that Grade 4 differed significantly from Grade 2 and Grade 1 (*p* = 0.006 vs. *p* = 0.002), showing a higher number of errors. Similarly, Grade 3 differed from Grade 1 (*p* = 0.049), also with an increased number of errors. In terms of the total errors, Grade 4 had significantly more errors compared to Grade 2 (*p* = 0.034) and Grade 1 (*p* = 0.003), while Grade 5 differed from Grade 1 (*p* = 0.043), and Grade 3 differed from Grade 1 (*p* = 0.044) (Table 4).

## 4. Discussion

Laparoscopic cholecystectomy is one of the most frequently performed procedures in general surgery; however, it is not exempt from complications, with bile duct injury remaining the most feared adverse event due to its significant clinical and medico-legal implications [1,2,3,4]. Ensuring procedural safety requires not only adequate surgical training and experience, but also the correct identification of anatomical structures, primarily through achievement of the Critical View of Safety (CVS), which remains the cornerstone of safe cholecystectomy [11,22].

In this context, the main contribution of the present study is to demonstrate that procedural safety and performance assessment are related but conceptually distinct domains. Procedural safety was evaluated through CVS achievement, whereas the Observational Clinical Human Reliability Analysis (OCHRA) system was used to characterize intraoperative technical deviations and error patterns. This distinction is essential, as OCHRA does not directly define whether a procedure is safe, but rather provides an objective framework for analyzing how the operation is performed and where deviations occur [16,17,23].

Our findings showed a high frequency of procedural errors, with a significant association between increasing intraoperative severity and a greater burden of errors, as well as a lower probability of achieving a satisfactory Doublet Photographic Score (DPS). Similarly, cases classified as Parkland grades 3–5 were associated with longer operative times, increased bleeding, and reduced rates of satisfactory CVS, consistent with previous studies validating the Parkland Grading Scale as a predictor of surgical difficulty and intraoperative risk [10,24]. From a clinical perspective, these results reinforce the complementary roles of the Parkland Grading Scale in stratifying operative difficulty and the CVS as the direct safety target during dissection.

OCHRA analysis revealed that most errors were procedural, particularly step repetition and the performance of additional, non-planned steps. Rather than reflecting technical incompetence, this pattern may represent adaptive behavior in response to complex intraoperative scenarios, where surgeons adjust their actions to maintain safety in the presence of inflammation, adhesions, or distorted anatomy. This interpretation is consistent with previous evidence demonstrating that situational awareness and intraoperative anticipation improve with experience and are critical determinants of surgical performance [15].

The positive correlation between intraoperative severity and procedural errors supports the role of OCHRA as a sensitive method for identifying early deviations that may precede adverse events [16]. In contrast, the lack of significant association with execution errors suggests that increasing anatomical complexity primarily affects procedural flow and decision-making rather than basic technical skills. This finding has relevant implications for surgical training, as it highlights the importance of focusing on decision-making processes and procedural strategy, particularly in difficult cases.

In this study, CVS was achieved in 54.7% of cases, with a progressive decrease observed as intraoperative severity increased. This finding is consistent with previous reports indicating that CVS may not always be safely achievable in high-complexity scenarios [25,26,27]. In such cases, insisting on obtaining CVS may be counterproductive and potentially increase the risk of bile duct injury. Therefore, alternative strategies such as subtotal cholecystectomy, intraoperative imaging, or timely conversion to open surgery should be considered integral components of safe cholecystectomy [5,22].

A relevant finding of this study is the absence of bile duct injuries despite the presence of procedural errors and intraoperative events such as bleeding and gallbladder perforation. This observation suggests that adherence to safety principles and appropriate intraoperative decision-making may compensate for technical deviations. Previous studies have shown that structured training in safe cholecystectomy improves the ability of surgeons to recognize and manage risk situations effectively [28,29,30].

A major strength of this study is the systematic video-based evaluation of real-world procedures across multiple surgical teams, allowing for an objective and reproducible assessment of surgical performance. Additionally, the integration of OCHRA with established safety measures such as CVS and the Parkland Grading Scale provides a comprehensive framework to analyze both technical performance and procedural safety.

From a clinical standpoint, the implementation of structured performance assessment through OCHRA may provide actionable insights beyond traditional outcome-based metrics. By identifying recurrent procedural deviations, this approach may facilitate targeted feedback, support individualized training strategies, and enhance intraoperative decision-making, particularly in complex cases [16,17]. Importantly, this potential clinical utility lies not in directly defining surgical safety, but in complementing established safety principles such as CVS by improving the consistency and quality of surgical execution.

Recent advances have explored the use of artificial intelligence (AI) models for intraoperative identification of anatomical landmarks during laparoscopic cholecystectomy as an adjunct to improve surgical safety. However, current evidence suggests that the performance and reliability of these systems remain limited in complex operative scenarios, particularly in cases with higher Parkland grades, where inflammation and distorted anatomy challenge automated recognition [14]. Moreover, most available studies remain exploratory and have not yet demonstrated consistent improvements in clinically relevant outcomes. In this context, structured human-centered approaches such as OCHRA continue to play a critical role in surgical assessment and performance analysis.

This study has several limitations. Its retrospective design may introduce selection bias, and the inclusion of multiple surgical teams with varying levels of experience may increase heterogeneity. Additionally, procedures converted to open surgery were excluded, potentially removing the most complex cases. Case selection depended on the availability and quality of video recordings, and video-based analysis may not fully capture all contextual factors influencing intraoperative decision-making. Therefore, these findings should be interpreted primarily as evidence on error patterns and performance assessment rather than as direct proof of improved clinical outcomes.

## 5. Conclusions

The findings of this study demonstrate that laparoscopic cholecystectomy safety is strongly influenced by intraoperative anatomical severity, which is associated with a higher frequency of procedural errors and a lower likelihood of achieving an adequate Critical View of Safety (CVS). In this context, the OCHRA system provides an objective and detailed framework for evaluating surgical performance by identifying intraoperative error patterns and their relationship with procedural complexity.

Importantly, OCHRA should be understood as a tool for performance assessment rather than a direct measure of surgical safety, which is primarily defined by established principles such as CVS. Its value lies in complementing these safety strategies by offering insights into the technical and cognitive aspects of surgical execution, thereby supporting improved intraoperative decision-making and greater consistency in surgical practice.

Future research should focus on prospective, multicenter studies evaluating the implementation of OCHRA as a feedback tool in surgical training programs [31] and its impact on surgical performance and patient outcomes. The potential role of emerging technologies such as artificial intelligence in video-based assessment warrants further investigation, although it remains beyond the scope of the present study.

## Figures and Tables

**Table 1:** Comparison of operative times according to Parkland Grading Scale. (*) indicates the group with the greatest difference identified by the Games–Howell post hoc analysis, and (†) by Tukey HSD (*p* < 0.05). Values are expressed as the mean ± standard deviation.

Operative Times	Parkland 1 *n* = 30	Parkland 2 *n* = 25	Parkland 3 *n* = 34	Parkland 4 *n* = 14	Parkland 5 *n* = 3	*p*
Total operative time	37.13 ± 18	43.92 ± 17.41	65 ± 32.34	76.21 ± 34.82 *	67 ± 28	<0.0001
Port insertion	4.13 ± 3.34	4.36 ± 2.58	5.97 ± 3.28	6 ± 4.97	3.67 ± 1.52	0.130
Cystic plate dissection	11.37 ± 10.33	13.52 ± 9.41	22.29 ± 15.69	26.57 ± 15.8 †	19.67 ± 7.57	0.001
Clipping and ligation	4.53 ± 3.10	7 ± 6.82	9.38 ± 13.92	10.21 ± 6.98	13 ± 8.54	0.154
Gallbladder dissection	7.23 ± 4.53	8.24 ± 4.19	12.26 ± 7.65	21.36 ± 13.92 *	18 ± 13.11	<0.0001
Hemostasis review	10.10 ± 6.75	11.48 ± 8.17	15.21 ± 10.22	11.71 ± 7.18	14.33 ± 2	0.164

**Table 2:** Comparison of intraoperative variables according to Parkland Grading Scale. (*) indicates statistically significant differences (*p* < 0.05) identified by Dunn’s post hoc analysis. DPS: Doublet Photographic Score; CVS: Critical View of Safety.

	Parkland 1 *n* = 30	Parkland 2 *n* = 25	Parkland 3 *n* = 34	Parkland 4 *n* = 14	Parkland 5 *n* = 3	*p*
Exposure of the cystic plate—clearly visible	26 (86.7%)	19 (76%)	10 (29.4%)	2 (14.3%)	0	<0.0001
Exposure of the hepatocystic triangle—clearly visible	27 (90%)	19 (76%)	10 (29.4%)	3 (21.4%)	0	<0.0001
Visualization of two structures entering the gallbladder—clearly visible	30 (100%)	21 (84%)	23 (67.6%)	11 (78.6%)	1 (33.3%)	<0.0001
DPS	6 (6–6) *	6 (4–6)	4 (3–6)	4 (2.5–4.25)	3 (0–4)	<0.0001
Satisfactory CVS	27 (90%)	18 (72%)	10 (29.4%)	3 (21.4%)	0	<0.0001
Gallbladder perforation	12 (40%)	13 (52%)	13 (38.2%)	10 (71.4%)	1 (33.3%)	0.244
Moderate-intense hemorrhage	5 (16.7%)	4 (16%)	15 (44.1%)	5 (35.7%)	2 (66.7%)	0.035

**Table 3:** Frequency of errors observed in the three stages of LC according to the OCHRA system classification. LC: laparoscopic cholecystectomy; OCHRA: Observational Clinical Human Reliability Analysis.

Pattern of Failure	Cystic Plate Identification and Dissection	Clipping and Division of the Cystic Artery and Duct	Dissection of the Gallbladder from the Hepatic Bed
Step is not done	3 (2.8%)	2 (1.9%)	2 (1.9%)
Step is partially completed	9 (8.5%)	10 (9.4%)	7 (6.6%)
Step is repeated	85 (80.2%)	89 (84%)	96 (90.6%)
Second step is done in addition	84 (79.2%)	87 (82.1%)	85 (80.2%)
Second step is done instead of first step	33 (31.1%)	29 (27.4%)	25 (23.6%)
Step is done out of sequence	13 (12.3%)	18 (17%)	15 (14.2%)
Step is done with too much force, speed, depth, distance, time, or rotation	4 (3.8%)	2 (1.9%)	4 (3.8%)
Step is done with too little force, speed, depth, distance, time, or rotation	0	0	1 (0.9%)
Step is done in wrong orientation, direction, or point in space	0	0	1 (0.9%)
Step is done on/with the wrong object (or plane)	0	0	1 (0.9%)

**Table 4:** Comparison of the number of errors according to Parkland Grading Scale. Values are expressed as median (interquartile range). (*) indicates statistically significant differences (*p* < 0.05) identified by Dunn’s post hoc analysis.

	Parkland 1 *n* = 30	Parkland 2 *n* = 25	Parkland 3 *n* = 34	Parkland 4 *n* = 14	Parkland 5 *n* = 3	*p* Value
Procedural errors in cystic plate dissection	2 (1.75–2)	2 (1.5–2)	2 (2–3)	3 (2–4) *	3(2.5–3.5)	<0.0001
Procedural errors in Clipping and division	2 (2–2)	2 (1.5–2)	2 (2–3)	3 (2–3.25) *	3 (3–3.75)	<0.0001
Procedural errors in gallbladder dissection	2 (2–2)	2 (2–2)	2 (2–3)	2.5 (2–3.25)	3 (2–3)	0.017
Total of execution errors	0 (0–0)	0 (0–0)	0 (0–0)	0 (0–0)	0 (0–1)	0.291
Total of procedural errors	6 (4.75–6)	6 (5–6)	6 (6–9)	8 (6–9.75) *	9(7.5–10)	<0.0001
Total of procedural and execution errors	6 (4.75–6) *	6 (5–6)	6 (6–9)	8.5 (6–9.75)	9 (8.25–10)	<0.0001

## Data Availability

Patient information is confidential. If needed, data can be requested from the corresponding author upon reasonable request.

## Abbreviations

The following abbreviations are used in this manuscript:

LC	Laparoscopic Cholecystectomy
SAGES	Society of Gastrointestinal and Endoscopic Surgeons
SC	Safe Cholecystectomy
CVS	Critical View of Safety
PGS	Parkland Grading Scale
OCHRA	Observational Clinical Human Reliability Analysis
DPS	Doublet Photographic Score
BMI	Body Mass Index
